# The Effect of a Modified Constant Flow Insufflation of Oxygen during Cardiopulmonary Resuscitation in a Rat Model of Respiratory Cardiac Arrest on Arterial Oxygenation, Alveolar Barotrauma, and Brain Tissue Injury

**DOI:** 10.1155/2020/8913571

**Published:** 2020-03-31

**Authors:** Yoonje Lee, Sang-hyun Lee, Hyuk Joong Choi, Jinkyu Park, Sejin Hwang, Tae Ho Lim, Changsun Kim

**Affiliations:** ^1^Department of Emergency Medicine, Gangnam Sacred Heart Hospital, Hallym University, Chuncheon-si, Republic of Korea; ^2^Department of Emergency Medicine, Hangang Sacred Heart Hospital, Hallym University, Chuncheon-si, Republic of Korea; ^3^Department of Emergency Medicine, Hanyang University Guri Hospital, Hanyang University, Seongdong-gu, Republic of Korea; ^4^Department of Cardiology, Hanyang University Medical Center, Hanyang University, Seongdong-gu, Republic of Korea; ^5^Department of Anatomy, College of Medicine, Hanyang University, Seongdong-gu, Republic of Korea; ^6^Department of Emergency Medicine, Hanyang University Medical Center, Hanyang University, Seongdong-gu, Republic of Korea

## Abstract

**Aim:**

Intermittent positive pressure ventilation (IPPV) can adversely affect cardiopulmonary resuscitation outcomes by increasing the intrathoracic pressure. Continuous flow insufflation of oxygen (CFIO) has been investigated as a potential alternative, but evidence supporting its superiority over intermittent positive pressure ventilation in cases of cardiac arrest is scant. The aim of the current study was to compare the effects of continuous flow insufflation of oxygen using a one-way valve during cardiopulmonary-resuscitation with intermittent positive pressure ventilation in a rat model of respiratory arrest.

**Methods:**

Male Sprague-Dawley rats weighing 400∼450 g (from minimum to maximum) were randomly assigned to either a sham, IPPV, or CFIO group (*n* = 10 per group). Respiratory arrest was induced by blocking the endotracheal tube. Arterial blood gas analysis was performed during cardiopulmonary resuscitation to compare the oxygenation levels. Tissues were then harvested to compare the degrees of pulmonary barotrauma and ischemic brain injury.

**Results:**

Return of spontaneous circulation was observed in 6/10 rats in the IPPV group and 5/10 in the CFIO group. During cardiopulmonary resuscitation, the mean PaO_2_ was significantly higher in the CFIO group (83.10 mmHg) than in the IPPV group (56.10 mmHg). Lung biopsy revealed more inflammatory cells and marked thickening of the alveolar wall in the IPPV group; the group also exhibited a higher frequency of neuroglial cells and apoptotic bodies of pyramidal cells, resulting from ischemic injury.

**Conclusion:**

In a rat model of respiratory arrest, CFIO using a one-way valve resulted in a greater level of oxygenation and less lung and brain injuries than with IPPV.

## 1. Introduction

A cardiopulmonary resuscitation (CPR) team performs positive pressure ventilation (PPV) using a bag-valve-mask, supraglottic airway, or endotracheal intubation combined with chest compression in cases of prehospital or in-hospital cardiac arrest (CA). During CPR, there must be sufficient venous return to allow ventricular filling during the relaxation phase to effect an adequate outflow during the active compression phase. However, although PPV may be an appropriate CPR technique, it may hinder venous return because it increases the ITP. Thus, inappropriate PPV may impair the quality of CPR [1].

Multiple studies have been conducted to resolve this problem. Kill et al. [2] introduced a method of synchronizing chest decompression and ventilation and reported that high oxygenation was achieved with this method in a porcine model of CA. Devices inhibiting the inspiration trigger during chest decompression, such as inspiratory impedance threshold device (ITD) and the Boussignac cardiac arrest resuscitation device (B-card), have also been investigated [3]. However, because the ITD still use a PPV technique. Also, in the B-card using the CFIO method, collision of oxygen inflow and exchanged gas outflow make pressure. This pressure can influence venous return and alveoli. Finally, brain injury caused by reduced brain blood flow might be a possibility due to reduced venous return, as well as reduced oxygen supply and increased ITP, causing pulmonary barotrauma.

In 2000, Saissy et al. [4] reported that the effects of continuous flow insufflation of oxygen (CFIO) were comparable to those achieved by intermittent positive pressure ventilation (IPPV) in cases of prehospital CA. In a study reported in 2004, investigating the application of CFIO and IPPV with endotracheal intubation in a porcine model of ventricular fibrillation (VF) CA, Steen et al. [5] reported that oxygenation and coronary perfusion pressure were significantly higher in the CFIO group.

In 2006, Bertrand et al. [6] reported that it is possible to deliver oxygen during CPR using CFIO in patients who have experienced prehospital CA. In a study involving patients with witnessed VF CA, Bobrow et al. [7] reported that patients who were delivered oxygen via CFIO were significantly more likely to survive and get discharged and exhibited better neurologic outcomes than patients who received PPV.

Notably, however, none of the above-menti–oned previous studies investigated whether CFIO could be applied in cases of respiratory CA. We hypothesized that CFIO using a one-way valve connected to an opening channel would be able to improve oxygenation than conventional IPPV, which would reduce ischemic brain injuries and pulmonary barotrauma. This hypothesis was investigated in the current study by comparing the effects of CFIO using a one-way valve, expected to reduce ITP during CPR, with conventional IPPV in a rat model of respiratory CA caused by oxygenation failure.

## 2. Methods

### 2.1. Study Design

This prospective randomized longitudinal rat study was approved by the institutional animal care and use committee at Hanyang University, South Korea (approval number 2016-0075A). The study was conducted at the Animal Study Laboratory of Hanyang University School of Medicine using Sprague-Dawley rats weighing 400–450 g. The rats had access to standard food and water ad libitum, and they were acclimatized to the environment for several days before the experiment. Cages were managed in accordance with the National Research Council standards.

### 2.2. Sample Size

The sample size was calculated based on the PCO_2_ levels of the IPPV and CFIO groups in the pilot study (PCO_2_ levels: IPPV group, 89.95 (86.70–91.63) mmHg and CFIO group, 99.90 (97.98–103.00) mmHg). The minimum sample size was 5 in each group, and an analysis with G-power 3.1.2® (Heine Heinrich University, Düsseldorf, Germany) with a 0.05 error level and 0.95 power was performed. The dropout rate was 30%, which accounted for the rats that were excluded from the experiment or could affect the experimental results before the end of the experimental protocol. The survival rate was estimated to be 50∼70% considering the histological examination of the surviving rats after CPR. Therefore, the final sample size was calculated as 10 rats for each group. When a rat died during preparation for the experiment or when serious problems arose that could affect the outcomes of the experiment, the rat was excluded from the experimental results.

### 2.3. Animal Study Protocol

During experiments, the temperature and humidity of the laboratory were consistently maintained at 24°C and 35%, respectively. To maintain body temperature, a surgical lamp was applied throughout all experiments, and the core temperature of all rats was maintained between 36°C and 37°C, the normal body temperature of rats. The rats were randomly divided into sham, IPPV, and CFIO groups via a computer program. Invasive blood pressure monitoring was performed in all rats via a femoral artery catheter while each rat was connected to a ventilator after the induction of deep anaesthesia via isoflurane with intramuscular injection of a mixture of Zoletil® (Virbac, Carros, France, zolazepam + ketamine, 30 mg/kg) and Rompun® (Bayel, Leverkusen, Germany, xylazine, 10 mg/kg) in a 2 : 1 ratio. The femoral artery catheter was also used for arterial blood gas analysis (ABGA). In the IPPV and CFIO groups, an intravenous line was inserted into the tail vein for epinephrine infusion during CPR.

Before inducing respiratory CA, 0.3 cc of arterial blood was collected from each rat to compare the baseline characteristics on ABGA among the three groups. After the ABGA in the sham group, while maintaining artificial ventilation, the 24 G catheter was removed from the femoral artery, and when the rats had recovered adequate spontaneous breathing, they were taken off the ventilator and placed in a new cage lined with warm cotton.

With reference to the respiratory CA model reported by Bai et al. [8] in 2015, the point of CA was set to the moment at which the mean arterial pressure dipped below 20 mmHg from 8 min after blocking of the endotracheal tube [3–6]. From this point, chest compression was begun at 200 pm for 2 min using metronome feedback, and one epinephrine dose (30 *μ*g/kg) was injected through the intravenous line. ROSC was checked after 2 min of chest compression. ROSC was determined based on a heart rate exceeding 200 bpm, palpation of the apical pulse, and invasive arterial blood pressure monitoring. Successful resuscitation was defined as ROSC that persisted for more than 10 min [9–13].

**Figure 1:**
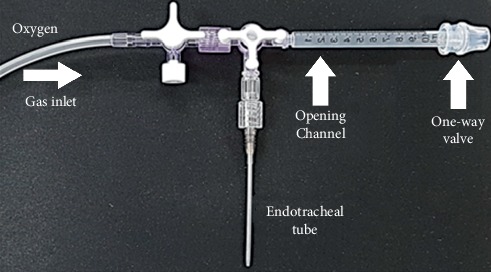
One-way valve with an opening channel.

During CPR, the IPPV group was put on PPV using a ventilator for rodents, and the CFIO group was administered oxygen via an endotracheal tube connected to a one-way valve (see Figure 1).

IPPV during CPR: using a rodent ventilator, the Inspira Advanced Safety Ventilator (HARVARD APPARATUS, Holliston, MA, USA) was set to provide one positive pressure breath with a tidal volume of 8 mL/kg animal weight for every four compressions (50 breaths/min).

CFIO during CPR: using a novel one-way valve connected with an opening channel, oxygen flow was provided through the gas inlet at 200 ml/kg/min to the one-way valve and to the endotracheal tube. During chest compression, gas from the lungs passes through the opening channel and opens the one-way valve. During chest decompression, oxygen flows and fills in the opening channel to the endotracheal tube and to all lung fields (see Figure 1).

After 1 min of CPR, a 0.3-cc arterial blood sample was taken for ABGA to compare the levels of oxygenation associated with the different ventilation methods. During CPR, isoflurane was not administered and the two groups received oxygen only.

**Figure 2:**
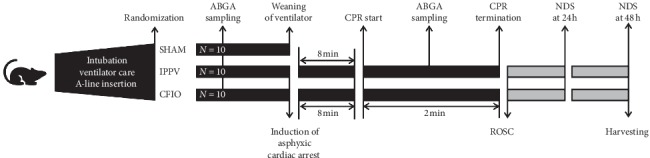
Animal study protocol. A-line, arterial line; IPPV, intermittent positive pressure ventilation; CFIO, continuous flow insufflation of oxygen; ABGA, arterial blood gas analysis; CPR, cardiopulmonary resuscitation; ROSC, return of spontaneous circulation; NDS, neurologic deficit score.

In successfully resuscitated rats, oxygen supply through the ventilator was resumed and ventilator care was restarted. Rats that exhibited ROSC were taken off the ventilator and moved to a new cage (see Figure 2). At 24 and 48 hours after resuscitation, quantitative neurologic assessments were performed with 21 items using the neurologic deficit score (NDS) system described by Jia et al. [14] (see Table 1).

### 2.4. Histologic Study Protocol

In accordance with the method described by Gage et al. [15], perfusion fixation was performed via intramuscular injection of Zoletil® and Rompun® in a 2 : 1 ratio 48 hours after resuscitation in a sedated state to prepare biopsy specimens. For lung biopsy, haematoxylin-eosin staining was performed. For brain biopsy, Nissl staining was performed to compare neuronal injuries.

### 2.5. Statistical Analysis

Test results were analysed using SPSS 21.0 software (IBM Analytics, IL, Chicago, USA). Although the results were normally distributed, the nonparametric Kruskal–Wallis test was used given the fact that there were 10 rats in each group. The sham group and two experimental groups were compared using the Kruskal–Wallis test. Pairwise comparisons were performed using the Mann–Whitney test with post hoc Bonferroni correction. From the total number of pyramidal cells observed at ×400 magnification in the Cornu-Ammonis 3 region of the hippocampus, which is vulnerable to hypoxic injuries, the percentages of stained apoptotic bodies and normal pyramidal cells were calculated for each slide six times for all rats. The results from the sham group were compared to those from the two experimental groups. As with the NDS, the percentages of all three groups were compared using the Kruskal–Wallis test, and paired comparisons (three comparisons) were performed using the Mann–Whitney test with post hoc Bonferroni correction (*p* < 0.017).

## 3. Results

Respiratory CA was induced in all rats of the two experimental groups, and 11 rats regained spontaneous circulation after CPR. Six of ten rats were in the IPPV group, and five of ten rats were in the CFIO group.

### 3.1. Baseline Characteristics of Each Group

There was no significant difference in body weight between the sham group (418.60 ± 2.55 g) and the two experimental groups (IPPV 417.30 ± 4.64 g, CFIO 417.90 ± 3.96 g). There was no significant difference in the time to respiratory CA between the IPPV group (500.90 ± 10.83 sec) and CFIO group (503.00 ± 10.08 sec), and there were no significant differences in the ABGA results (pH, PaCO_2_, PaO_2_, HCO_3_^−^, base excess, and lactate) before CA in these two groups (see Table 2).

### 3.2. Results of ABGA Performed during CPR

There were no significant differences in the arterial blood pH, HCO_3_^−^, base excess, or lactate during CPR between the two experimental groups (*p* > 0.05 for all). However, the CFIO group had significantly higher PaCO_2_ (99.90 mmHg) and PaO_2_ (83.10 mmHg) than the IPPV group (PaCO_2_ 89.95 mmHg, PaO_2_ 56.10 mmHg; *p* < 0.001 for both comparisons) (see Table 3).

### 3.3. Neurologic Outcome Assessment

**Figure 3:**
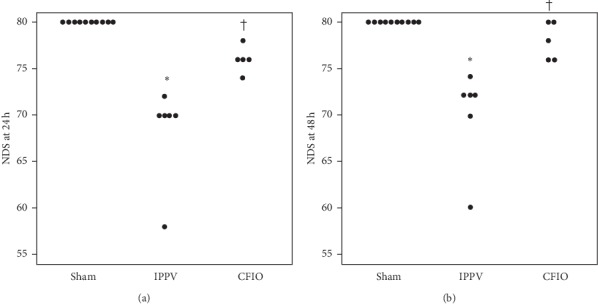
Neurologic deficit scores 24 hours (a) and 48 hours (b) after the return of spontaneous circulation in a rat model of respiratory arrest. Sham group (*n* = 10), IPPV group (*n* = 6), CFIO group (*n* = 5), and ^*∗*^*p* < 0.01 compared to the sham group; ^†^*p* < 0.017 compared to the IPPV group. NDS, neurologic deficit score; IPPV, intermittent positive pressure ventilation; CFIO, continuous flow insufflation of oxygen.

NDS 24 hours after ROSC was 70 in the IPPV group, which was significantly lower than that in the sham (80; *p* < 0.001) and CFIO (76; *p*=0.004) groups; however, the sham group did not differ significantly from the CFIO group (*p*=0.081). NDS 48 hours after ROSC was 74 in the IPPV group, which was significantly lower than that in the sham (80; *p* < 0.001) and CFIO (78; *p*=0.001) groups; however, the sham group did not differ significantly from the CFIO group (*p*=0.398 (see Figure 3).

### 3.4. Lung Biopsy Results

**Figure 4:**
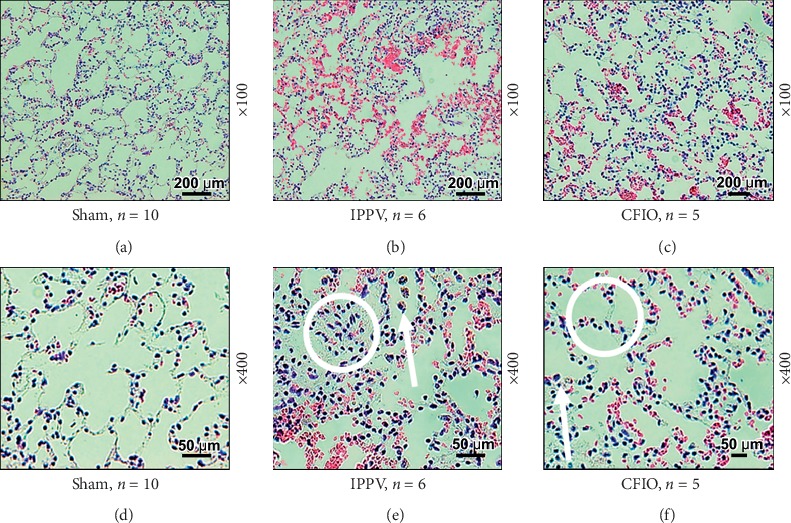
Histologic staining of lung tissue with haematoxylin-eosin 48 hours after the return of spontaneous circulation. Alveolar wall thickening was greater in the IPPV group than in the CFIO group, and there were more inflammatory cells. White arrows are pointing at inflammatory cells, and white circles indicate thickening of the alveolar wall. IPPV, intermittent positive pressure ventilation; CFIO, continuous flow insufflation of oxygen.

Lung biopsy specimens were haematoxylin-eosin-stained for comparison. Compared with the sham group, the two experimental groups exhibited thickening of the alveolar wall, caused by barotrauma and multiple inflammatory cells. Most cells were macrophages and were observed in the thickened alveolar wall and inside the alveoli. These observations were more marked in the lung samples of the IPPV group than in the CFIO group (see Figure 4).

### 3.5. Brain Biopsy Results

**Figure 5:**
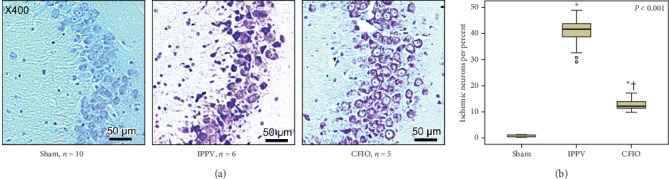
Histologic examination of hippocampal Cornu-Ammonis 3 regions with cresyl violet staining 48 hours after the return of spontaneous circulation. (a) Representative images of cresyl violet staining at 400 × magnification. There were more apoptotic pyramidal cells and neuroglial cells in the IPPV group than in the CFIO group. (b) Boxplots representing the proportions of ischemic neurons per total number of neurons. ^*∗*^*p* < 0.001 compared to the sham group; ^†^*p* < 0.001 compared to the IPPV group. IPPV, intermittent positive pressure ventilation; CFIO, continuous flow insufflation of oxygen.

In the Nissl-stained brain tissue specimens, the IPPV group exhibited a higher percentage of apoptotic pyramidal bodies caused by ischemic injuries and an elevated count of neuroglial cells, which respond to nerve injuries, compared with the CFIO group (Figure 5(a)).

### 3.6. Percentage of Ischemic Injured Brain Cells and Normal Pyramidal Bodies

The percentages of apoptotic bodies caused by ischemic injury in each group were calculated and compared. In the sham group, these percentages were significantly lower than they were in both the IPPV group (41.01 ± 4.62, *p* < 0.001) and the CFIO group (12.73 ± 2.09, *p* < 0.001), and the percentages in the CFIO group were significantly lower than those in the IPPV group (*p* < 0.001) (Figure 5(b)).

## 4. Discussion

The major findings of the current study were that the CFIO method using a one-way valve connected to an opening channel led to a greater level of oxygenation and lower pulmonary barotrauma and hypoxic brain injury than those produced by the conventionally recommended IPPV method in a rat model of respiratory CA.

Research on the possibility of delivering oxygen via CFIO began in the 1980s. In 1982, Lehnert et al. [16] first reported the possibility of oxygenation via CFIO in a canine apnoea model. In 1991, Brochard et al. [17] reported that CFIO may be used in limited situations wherein the patient must be taken off artificial respiration, such as during suctioning through an endotracheal tube. In 2004, Meggs et al. [18] reported that pigs in a porcine apnoea model were able to survive without respiratory effort for an average of 75 min while CFIO was maintained. Several subsequent studies investigating the feasibility of CFIO in CPR followed on the basis of these initial studies [4–7].

In a study on prehospital CA patients published in 2000, Saissy et al. [4] reported that CFIO yielded comparable effects to those of IPPV, as indicated by significantly similar ABGA results associated with the two methods. However, ABGA was performed after ROSC in that study; therefore, the parameters were not accurate representations of oxygenation during CPR. Furthermore, the study did not analyse differences in brain or lung injuries or neurologic outcomes associated with each method of ventilation.

In 2004, Steen et al. [5] reported that CFIO led to a significantly higher level of oxygenation and higher coronary perfusion pressure than IPPV in a porcine model of VF CA, but the study was limited to VF CA induced in a sufficiently oxygenated state and did not analyse hypoxic brain injuries or differences in lung barotrauma or oxygenation between the ventilation methods.

In 2006, Bertrand et al. [6] reported that CFIO delivered sufficient oxygen in patients with prehospital CA, but they also reported that the patients had poor outcomes. Notably, however, the grounds for claiming that a higher level of oxygenation was induced by CFIO in that study was that there was a significantly higher number of patients with oxygen saturation of greater than 70% during CPR in the CFIO group, as opposed to measuring oxygen partial pressure—an index for oxygenation—via ABGA during CPR. Furthermore, although differences between the thoracic injuries associated with the two ventilation methods were investigated in that study, it was simply stated that the incidence of rib fracture was higher in the IPPV group than in the CFIO group. Pulmonary barotrauma was not evaluated via biopsy. On the basis of this additional finding, Bertrand et al. [6] speculated that PPV would increase ITP, and when combined with the elevated pressure caused by chest compression, it may increase the risk of rib fracture.

In 2009, Bobrow et al. [7] compared patients with witnessed prehospital VF CA who were either provided oxygen via a mask or administered PPV using a bag valve mask and reported that the former group had better neurologic outcomes. However, that study was limited, in that it involved VF, in which chest compression and defibrillation are more important than oxygenation.

These abovementioned studies evaluating the effects of CFIO [4–7] were focused on human patients or animal models of VF CA, in which the importance of oxygenation is relatively lower than it is for respiratory CA. Moreover, the CFIO methods used in these studies involved the delivery of oxygen using multiple channels or via a general endotracheal tube or mask. In contrast, the present study utilized a modified CFIO technique in which the inspiratory flow into the endotracheal tube is strengthened by closing the valve to allow oxygen accumulation in the opening channel of the tube. The valve is then opened in response to elevated pressure in the opening channel during chest compression, so as not to increase ITP. Furthermore, the present study utilized a rat model of respiratory CA such that oxygenation is important for recovery. The oxygenation effects of two different ventilation methods were investigated by performing ABGA during CPR. And an alveolar barotrauma and hypoxic brain injury associated with the two methods also were assessed via histological evaluation of lung and brain biopsy from survived rats.

Although the current study used a rat respiratory CA model, oxygenation during CPR was significantly higher in the CFIO group than in the IPPV group, which is similar to previous results derived from VF patients and animal models [4–7]. Haemodynamic indicators such as cardiac output could not be measured in the current study because we used rats rather than pigs, but the associated findings were the same in both studies.

In the present study, for the CFIO method, an endotracheal tube connected to a one-way valve and an opening channel were utilized. This new CFIO method was designed such that endotracheal inspiratory flow is strengthened during chest decompression by the oxygen accumulated in the opening channel as a result of the closed valve. Furthermore, during chest compression, there is no collision between oxygen flowing toward the valve and gas being pushed out because the valve is not directly located within the channel connected to the trachea. Instead, the outflow of gas from the airway is actually promoted because the valve is opened due to the pressure in the opening channel, and the oxygen flow is directed toward the valve. These features are thought to reduce ITP, and consequently, the CFIO group exhibited less severe alveolar damage and hypoxic brain injury compared with the IPPV group.

The current study has some limitations. First, the body temperatures of the surviving rats were not monitored after they were placed back into a cage. Rats tend to exhibit spontaneous reduction in body temperature when recovering from CA [10], but the consequent effects were not considered in the present study. Second, respiratory CA was induced while providing 100% oxygen, so the models utilized may differ from real-life respiratory CA occurring amid exposure to 21% oxygen, the oxygen concentration in normal air. Third, CPR was only performed for 2 min after the induction of CA, in an attempt to eliminate the effects of the duration of CPR while investigating differences in pulmonary and brain tissue injuries according to the ventilation method. This may have affected the histological outcomes. Fourth, we did not check the airway pressure during CPR in each group. A continuous flow of oxygen toward the lower airway is likely to create a positive pressure. According to the study by Steen et al. (2004), the CFIO group performed better in terms of oxygenation than the IPPV group, but a higher airway pressure during CPR was reported than the IPPV group. This may be due to a positive pressure created by the continuous flow of oxygen toward the lower airway. And they did not use one-way valves, nor did they use opening channels to control the tidal volume induced during decompression. However, in this study, the CFIO group showed less damage due to pressure, according to alveolar histological findings. Further studies are needed to clarify the above findings, and we aim to conduct a large animal experiment in the future. Finally, the study used rat models, so the results cannot be directly extrapolated to humans. The results of the study are similar to those of some previous human studies [4–7]; however, they shed light on the potential usefulness of CFIO incorporating a one-way valve with an opening channel during CPR by comparing the brain and lung biopsy results associated with different ventilation methods.

Additional large animal studies are needed to compare the haemodynamic outcomes of three different ventilation methods using the inspiratory ITD, B-card, including the device used in the present study. Such studies may facilitate the development of new artificial ventilation methods or devices that address the problems associated with the long-recommended IPPV method during CPR, e.g., the need for a person to be in charge of ventilation during CPR, possibility of tissue damage caused by PPV, and reduced quality of CPR due to PPV.

## 5. Conclusion

In a rat model of respiratory CA, the CFIO method using a one-way valve designed to lower ITP during CPR resulted in a higher level of oxygenation with lower incidences of lung and brain injuries compared with the IPPV method. These protective effects on the lung and brain may be associated with the one-way valve, which was designed to induce a greater reduction in barotrauma of the lung and result in higher oxygenation than that in the IPPV method.

## Figures and Tables

**Table 1:** Neurologic deficit scoring system.

Items (score)		Scoring system		
General behaviour deficit (0–19)	Consciousness Arousal (eye opening) Respiration	Normal (10) Spontaneous (3) Normal (6)	Stupor (5) Pain (1) Abnormal (3)	Comatose (0) No (0) Absent (0)

Brain stem function (0–21)	Olfaction	Present (3)		Absent (0)
	Vision	Present (3)		Absent (0)
	Pupillary reflex	Present (3)		Absent (0)
	Corneal reflex	Present (3)		Absent (0)
	Startle reflex	Present (3)		Absent (0)
	Whisker stimulation	Present (3)		Absent (0)
	Swallowing	Present (3)		Absent (0)

Motor and sensory (0–12)	Strength, right	Normal (3)	Stiff/Weak (1)	Paralyzed (0)
	Strength, left	Normal (3)	Stiff/Weak (1)	Paralyzed (0)
	Pain withdrawal, right	Brisk (3)	Weak (1)	No (0)
	Pain withdrawal, left	Brisk (3)	Weak (1)	No (0)

Behaviour (0–18)	Gait coordination	Normal (3)	Abnormal (1)	Absent (0)
	Balance on beam	Normal (3)	Abnormal (1)	Absent (0)
	Righting reflex	Normal (3)	Abnormal (1)	Absent (0)
	Negative geotaxis	Normal (3)	Abnormal (1)	Absent (0)
	Visual placing	Normal (3)	Abnormal (1)	Absent (0)
	Turning alley	Normal (3)	Abnormal (1)	Absent (0)

Seizures (0–10)	Seizures	No (10)	Focal (5)	General (0)

**Table 2:** Baseline characteristics of the experimental rats, all of which were male and approximately 15 weeks old.

	Sham (*n* = 10)	Group IPPV (*n* = 10)	Group CFIO (*n* = 10)	*p* value
Age		15 weeks old		
Sex		Male		
Weight, g	419.00 (415.75–421.00)	417.50 (413.25–421.25)	418.00 (414.50–421.25)	0.86
Arrest induction time, s	NA	499.50 (491.25–510.00)	500.00 (495.75–508.00)	0.66
pH	7.32 (7.29–7.34)	7.33 (7.31–7.35)	7.32 (7.29–7.34)	0.79
PCO_2_	32.00 (30.25–33.78)	31.55 (30.25–34.43)	30.15 (29.63–32.13)	0.19
PO_2_	121.15 (119.18–125.45)	121.70 (116.85–127.68)	120.65 (118.95–125.65)	0.98
HCO_3_^−^	30.10 (29.33–30.90)	29.65 (28.98–30.23)	29.10 (28.60–29.65)	0.31
Base excess	−1.55 (−1.8–−1.2)	−1.3 (−1.93–1.08)	−.3 (−1.43–−1.18)	0.67
Lactate	2.00 (1.78–2.10)	2.10 (2.00–2.30)	2.05 (1.98–2.13)	0.31

Kruskal-Wallis analysis, median (IQR), *p* < 0.05 was considered statistically significant. IPPV, intermittent positive pressure ventilation; CFIO, continuous flow insufflation of oxygen; NA, not applicable.

**Table 3:** Comparisons of arterial blood gas parameters in two experimental groups 1 min after the start of cardiopulmonary resuscitation in a rat model of respiratory arrest.

	IPPV group	CFIO group	*p* value
pH	7.01 (6.98–7.05)	6.99 (6.97–7.00)	0.06
^*∗*^PCO_2_	89.95 (86.70–91.63)	99.90 (97.98–103.00)	<0.001
^*∗*^O_2_	56.10 (54.68–58.25)	83.10 (81.05–87.50)	<0.001
HCO_3_-	23.90 (22.58–24.95)	24.10 (23.10–24.98)	0.59
Base excess	−4.70 (−6.35–−4.15)	−4.40 (−5.13–−4.08)	0.40
Lactate	4.55 (4.10–5.10)	4.85 (4.30–5.15)	0.45

Mann–Whitney test, median (IQR), *p* < 0.05 was considered statistically significant. ^*∗*^Statistical significant. ABGA, arterial blood gas analysis; CPR, cardiopulmonary resuscitation; IPPV, intermittent positive pressure ventilation; CFIO, continuous flow insufflation of oxygen.

## Data Availability

The data used to support the findings of this study are available from the corresponding author upon request.
